# Quantitative Validation of the *n*-Butanol Sniffin’ Sticks Threshold Pens

**DOI:** 10.1007/s12078-014-9168-1

**Published:** 2014-04-29

**Authors:** Melanie Y. Denzer, Stefan Gailer, David W. Kern, L. Philip Schumm, Norbert Thuerauf, Johannes Kornhuber, Andrea Buettner, Jonathan Beauchamp

**Affiliations:** 1 Department of Chemistry and Pharmacy, Emil Fischer Center, Friedrich-Alexander-Universität Erlangen-Nürnberg, Henkestraße 9, 91054 Erlangen, Germany; 2 Department of Psychiatry and Psychotherapy, Friedrich-Alexander-Universität Erlangen-Nürnberg, Schwabachanlage 6, 91054 Erlangen, Germany; 3 Department of Sensory Analytics, Fraunhofer Institute for Process Engineering and Packaging IVV, Giggenhauser Strasse 35, 85354 Freising, Germany; 4 Department of Comparative Human Development, and The Institute for Mind and Biology, The University of Chicago, 940 E 57th St., Chicago, IL USA; 5 Department of Health Studies, The University of Chicago, 5841 South Maryland Ave., MC2007, Chicago, IL USA

**Keywords:** *n*-Butanol, Olfaction, PTR-MS, Sniffin’ Sticks, Odor threshold test

## Abstract

Odorant pens are used by medical practitioners and researchers to assess olfactory dysfunction. Despite their routine use, there are currently no data on the gas-phase odorant concentrations released from the pen tips or whether these concentrations scale linearly with the aqueous-phase concentrations inside the pens. The commercially available Sniffin’ Sticks odor threshold test containing *n*-butanol was chosen for evaluation. The gas-phase concentration of *n*-butanol at the tip of each pen was measured directly in a new set of pens via proton-transfer-reaction mass spectrometry (PTR-MS). Measurements were additionally made on the same pens after 6 months and two older pen sets, namely a 3-year-old (used) and 4-year-old (new) set. Furthermore, application-related tests were made to determine the performance of the pens during routine use and under stress. These data demonstrate that the gas-phase *n*-butanol concentrations of the threshold pens are linear over the entire set, both for brand-new pens and 6 months later; this reflects the expected performance that was previously only assumed. Furthermore, the application–simulation tests demonstrated a good performance of the pens when used according to their intended protocol. Measurements of the older pen sets suggest that storage conditions are more critical than usage for pen stability. The present findings confirm that the *n*-butanol odorant pens are an appropriate tool for threshold testing, provided they are stored and handled correctly.

**Figure:**
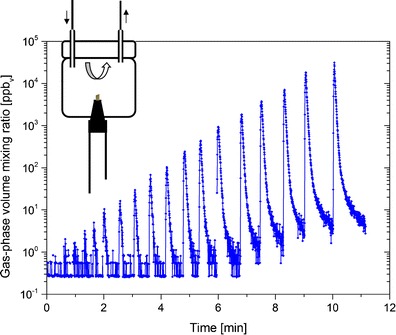
ᅟ

ᅟ

## Introduction

Olfactory dysfunction is a condition in which the sense of smell is impaired or even completely lost and can accompany or manifest after illness, head trauma, or diverse pathologies such as Alzheimer’s, Parkinson’s, and Huntington’s diseases, multiple sclerosis, amyotrophic lateral sclerosis, epilepsy, and schizophrenia (Doty 1997; McKeith et al. 2003; Zaccai et al. 2006; Haehner et al. 2009a), as well as mild cognitive impairment (Djordjevic et al. 2008; Lehrner et al. 2009; Sohrabi et al. 2012). It has been estimated to affect some 20 % of populations (Brämerson et al. 2004; Vennemann et al. 2008) and can lead to a marked reduction in quality of life, as well as being linked to depression and obesity (Hoover 2010). Accurate evaluation and diagnosis of a loss in the sense of smell is therefore imperative to enable treatment or counseling, as necessary.

Several tests have been designed to identify olfactory dysfunction on an objective basis. Simmen and co-workers developed a test based on diskettes containing a range of different odorants that are released upon opening and reported its applicability for screening olfactory dysfunction (Simmen et al. 1999). Doty and others (1984) developed the UPSIT based on scratch-and-sniff odor panels that can be used for testing a wide variety of olfactory disorders. The CCCRT consists of an identification test that includes ten test substances and an *n*-butanol threshold test for the examination of olfactory dysfunctions (Cain et al. 1988). Hummel and colleagues (1997) created a test battery based on odorant pens, known as Sniffin’ Sticks. The Sniffin’ Sticks include a threshold test, a discrimination test, and an identification test to assess multiple components of olfactory dysfunction. The *n*-butanol odor threshold test is widely used for the evaluation of olfactory sensitivity and has been successfully employed in multiple research fields to investigate the influence of age and gender on olfactory sensitivity (e.g., Hummel et al. 2007; Markovic et al. 2007; Thuerauf et al. 2009).

The Sniffin’ Sticks test battery has been validated from a clinical approach (Kobal et al. 2000; Albrecht et al. 2008; Tekeli et al. 2013), but the pens have not been comprehensively evaluated on a chemical–analytical basis to date. Although routinely used to assess dysfunction, the linearity of the odor concentrations emitted from the pen tips of the *n*-butanol threshold pens is currently unknown. The aim of the present work is to investigate the linearity of the *n*-butanol gas-phase concentrations released from the tips of the Sniffin’ Sticks threshold pens and thereby evaluate the applicability of this test for olfactory sensitivity testing.

## Materials and Methods

### Materials

Three different threshold evaluation sets of *n*-butanol Sniffin’ Sticks (Burghart Messtechnik GmbH, Wedel, Germany) were assessed: (1) a new, unused set (referred to as *new*), (2) a 3-year-old set that had been used previously in a research setting (*3 years, used*) (Kern et al. 2010), and (3) a 4-year-old unused set (*4 years, new*). Each set consisted of 16 different pens in a 1:2 volume per volume (*v/v*) aqueous dilution series at concentrations ranging from 4 % (designated as pen no. 1) to 1.2 ppm_v_ (parts per million, by volume) (pen no. 16) (Hummel et al. 1997). The full concentration series is listed in Table 1. In addition, the *new* and *3 years, used* sets each had an 8 % *v/v n*-butanol pen (pen no. 0), which was a special fabrication from the manufacturer for a large population study (Kern et al. 2014).

**Table 1:** Aqueous-phase concentrations of *n*-butanol in each pen of the Sniffin’ Sticks threshold evaluation set according to the manufacturer.

Pen no.	0 ^a^	1	2	3	4	5	6	7	8	9	10	11	12	13	14	15	16
Conc. (*v/v*)	8	4	2	1	0.5	0.25	0.125	0.063	0.031	0.016	78.1	39.1	19.5	9.77	4.88	2.44	1.22
Unit	%	%	%	%	%	%	%	%	%	%	ppm_v_	ppm_v_	ppm_v_	ppm_v_	ppm_v_	ppm_v_	ppm_v_

*ppm*_*v*_ parts per million, by volume

^a^Pen no. 0 was available only in the *new* and *3 years, used* pen sets (see text)

*n*-Butanol is a substance with a low vapor pressure (6.6 hPa at 20 °C) (Eickmann 2008) that is soluble in water up to 7.9 g per 100 g water at 20 °C (Rauscher et al. 1996). The volatility of *n*-butanol relative to water is 0.7 (Eickmann 2008), which means that water evaporates at a slightly faster rate than *n*-butanol.

### Analytical Tools and Sampling Setup

A high-sensitivity proton-transfer-reaction mass spectrometer (PTR-MS; IONICON Analytik GmbH, Innsbruck, Austria) was used for the present assessments. The operating principles of the instrument have been described in detail in the literature (Hansel et al. 1995; de Gouw et al. 2003; de Gouw and Warneke 2007) and will not be repeated here. In brief, the technique is based on soft chemical ionization via proton transfer reactions from hydronium reagent ions (H_3_O^+^) to ionize and quantitatively detect volatile organic compounds (VOCs), including odorants such as *n*-butanol. A distinct advantage of this method over conventional systems for VOC detection, such as GC-MS, is its online detection capability, i.e., gas can be continuously sampled without the need for sample pre-treatment, enabling VOC detection at a high frequency (in quasi real-time). The PTR-MS reaction chamber was operated at a voltage of 600 V, a pressure of 2.2 mbar, and a temperature of 60 °C, which produced an electric field strength to buffer gas number density ratio (*E/N*) of 132 Td (1 Townsend (Td) = 10^−17^ V cm^2^). The sampling inlet consisted of a Silcosteel™ capillary (Restek, Bellafonte, PA, USA) of ∼1 m length, with an inner diameter (ID) of 0.1″ and an outer diameter (OD) of 1/16″. This inlet was heated to 70 °C and had a sample flow rate of 58 standard cubic centimeters per minute (sccm).

**Fig. 1:**
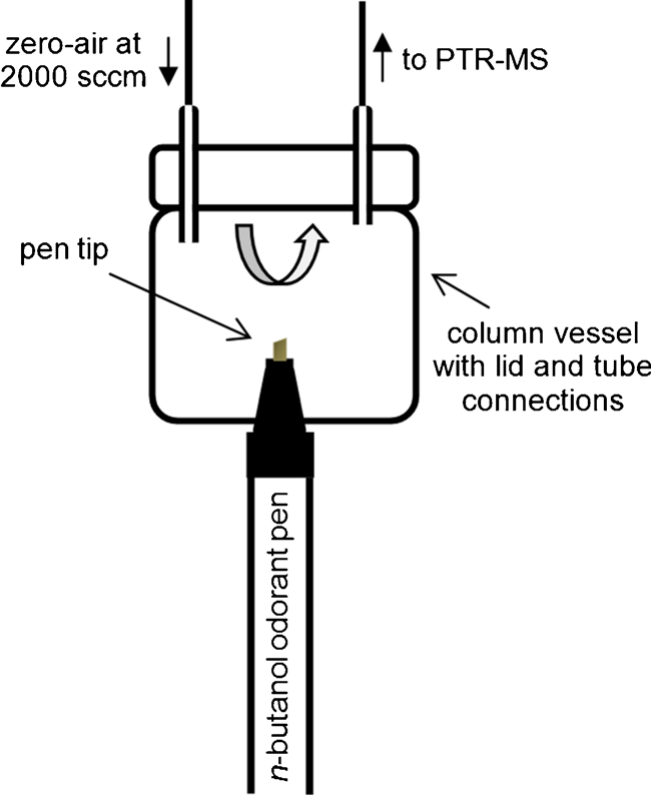
Schematic of the measurement setup

Odor release from the tips of the Sniffin’ Sticks pens was measured via a perfluoroalkoxy (PFA; Teflon®) column component vessel of 60 mL volume (AHF analysentechnik GmbH, Tübingen, Germany) and detachable lid with two 1/8″ ID fittings. A 12-mm-diameter hole was drilled into the base of the column for introduction of the tip of each Sniffin’ Sticks pen. A schematic of the measurement setup is given in Fig. 1. A carrier gas flow of VOC-free dry air—known as zero-air—at a flow rate of 2,000 sccm was generated by an advanced-model gas calibration unit (GCU-a; IONICON Analytik GmbH; see also Beauchamp et al. (2013)) and was used to flush the column (via one connection port on the column lid). The PTR-MS inlet capillary was connected to the column (via the second connection port on the lid) via a short length of 1/8″ OD, 1/16″ ID PFA tubing to allow continuous measurement of the sample gas within the column.

During the assessments, the cap of each pen was removed and the tip was fully inserted into the 12-mm orifice at the base of the column such that the pen tip was approximately 20 mm inside. The high flow rate of zero-air compared with the relatively small column volume (2,000 cm^3^ min^−1^ flow versus 60 cm^3^ volume) ensured rapid flushing of the vessel (equivalent to an air exchange interval of 1.8 s) that reduced the dead-space volume within the column. Furthermore, although typical sniff flows are considerably higher (∼10,800 sccm; see, e.g., Hahn et al. (1993)), data here are reported as volume mixing ratios (VMRs), thus the concentration ratios determined from one pen to the next are proportional, regardless of the flow-rate or volume of gas sampled. An electronic analog signal connected to the PTR-MS was manually triggered using a custom-built 0–5 V switch to flag the period of pen presentation within the PTR-MS dataset; this aided data post-processing by allowing data to be filtered according to the presence/absence of a pen during the continuous analysis.

The PTR-MS was set to measure in selected ion monitoring mode. Preliminary measurements of pure *n*-butanol indicated that the ion at a mass-to-charge ratio *m/z* 57 was the predominant analyte product ion of this compound under the given settings. The instrument was set to measure a total of five ion channels—*m/z* 21 (the isotopolog of the reagent hydronium), *m/z* 37 (the water cluster of hydronium), *m/z* 39 (the isotopolog of *m/z* 37, as well as a product ion (fragment) of *n*-butanol), *m/z* 57 (the most abundant analyte of *n*-butanol, as well as the isotopolog of the hydronium water cluster at *m/z* 55), and *m/z* 59 (a fragment ion of propylene glycol, which is often used as a carrier medium in other odorant pens but was not used further in the data evaluation of the present study). Each ion was measured with a dwell time (i.e., detection time) of 50 ms, resulting in a cycle time (measurement frequency) of 250 ms (4 Hz).

### Measurement Protocol

The Sniffin’ Sticks odor threshold evaluation test set is administered as an olfactory evaluation on the principle that *n*-butanol is released from the tip of the pen upon presentation below a subject’s nose. The amount of *n*-butanol present in the air surrounding the tip of a pen can be quantified as a gas-phase concentration and is generated via physical evaporation of *n*-butanol from its aqueous-phase within the storage medium of the pen. A dynamic equilibration exists between the aqueous-phase odorant concentration in the pen and its gas-phase concentration in air at the tip of the pen.

A series of assessment procedures was carried out to simulate different situations and uses of the pens, as outlined in the following. Measurements of individual Sniffin’ Sticks pens were made in triplicate for each of the tests described below, unless otherwise stated.

#### Linearity Assessments

**Fig. 2:**
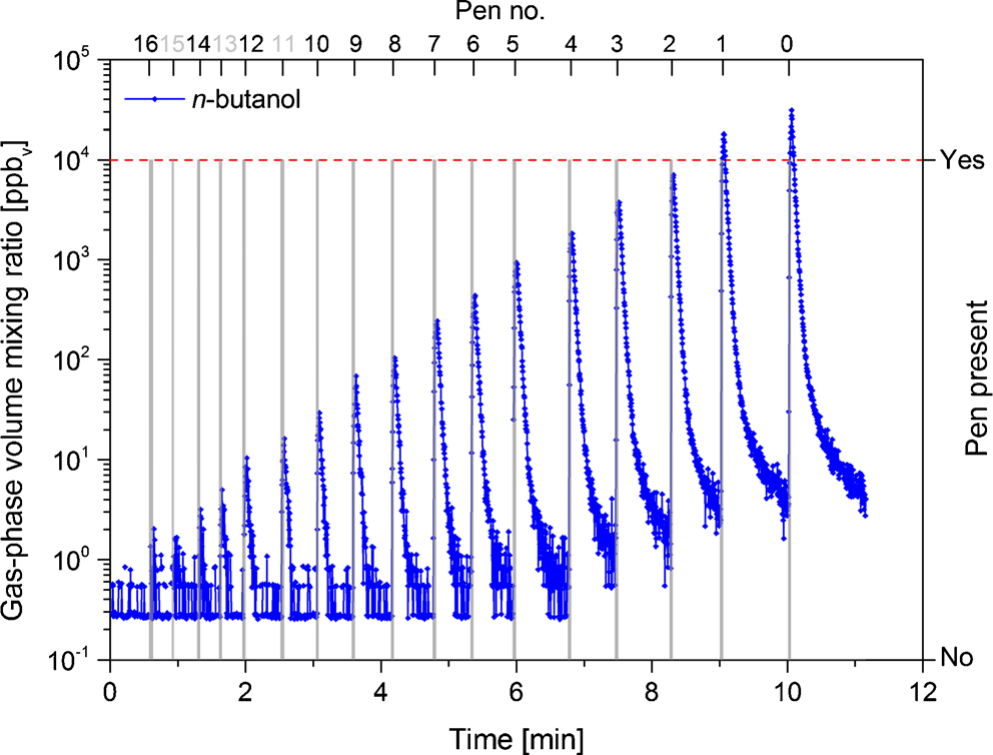
Time series of *n*-butanol concentrations (detected by PTR-MS at *m/z* 57) during the first measurement of the *new* odorant pen set. The presence of a pen is depicted with *vertical columns that reach the dashed line*. Note the use of a logarithmic scale

Odorant pens were measured one at a time, with the PTR-MS instrument set to measure continuously throughout this procedure as outlined above. Using the experimental setup described above, the cap of a pen was removed and the pen tip was inserted into the orifice at the base of the column for 3 s. Measurements started with the pen containing the lowest concentration (pen no. 16), followed by the next highest concentration (pen no. 15), and so on up to pen no. 1 (or pen no. 0, for the *new* and *3 years, used* sets). The raw data of a measurements series are shown in Fig. 2. After completing this test with the entire dilution series, the measurements were repeated (twice) to provide triplicate data for each odorant pen. In addition, assessment of the *new* set was repeated after 6 months (test condition referred to as *6 months, used*) to investigate the pens after their recommended manufacturer’s ‘use-by’ period of half a year. In this manner, the linearity of the gas-phase *n*-butanol concentration emitted from the pen tips over the entire set of pens could be assessed.

#### Application Test

In a clinical setting, two successive presentations of an individual pen with successful detection are necessary to consider a concentration as correctly detected. Such repeated use of a single pen in close succession poses a potential problem of a possible depletion of *n*-butanol released from the pen. To gather more information about the concentration stability of gas-phase *n*-butanol released from a single pen during a typical odor threshold assessment procedure, an application test was carried out to simulate repeated use of the odorant pens. This application test was made only with the *new* odorant pen set.

Starting with the lowest concentration (pen no. 16), each pen was uncapped and directly measured for 3 s, then recapped for 20 s, and subsequently uncapped again and measured for another 3 s. This procedure was repeated six times in succession for each pen before moving on to the next.

#### Stress Tests

Tests were carried out to evaluate *n*-butanol emissions when pens were stressed beyond their typical clinical use. These tests were carried out using only the *new* pen set, after completing the application test (see “Application Test” section).

In a first test, the cap of each pen was opened for 2 s and closed for 1 s, 20 times in succession (test condition referred to as *recapped*). The pen tip was then measured as described earlier, measuring pen no. 16 through to pen no. 1 (see “Linearity Assessment”). This enabled a comparison of gas-phase *n*-butanol concentrations to be made with the values measured during the linearity assessment of the same pen set (*new*), thereby providing an indication of how such stress might affect the pens.

A second stress test was used to simulate operator misuse. In this test, the cap of a pen was removed and the pen was left uncapped for 1 min before measurement (test condition referred to as *uncapped*). Again, the pen tip was measured as described earlier, measuring pen no. 16 through to pen no. 1. Furthermore, in order to assess the potential replenishment after such an event, the pens (nos. 16 to 1) were assessed again after a regeneration period of 4 h with closed caps (test condition referred to as *regeneration*). Assessment via PTR-MS was conducted in a similar manner as previously described (see “Linearity Assessment”).

### PTR-MS Data Evaluation

The raw PTR-MS signal intensity (in cps) of *n*-butanol at *m/z* 57 was post-processed as follows: The background “noise” on this ion channel was calculated as a mean value from the initial measurement cycles of each experimental run in the absence of a stimulus, i.e., prior to presentation of the pens (measurement of zero-air via the sampling column with no odorant pen present). This noise relates to the ^18^O-isotope of the (H_2_O)_2_⋅H_3_O^+^ water cluster (present at *m/z* 55, albeit at very low intensity for the instrument settings used here), as well as to residual *n*-butanol adsorbed to the column and tubing inner surfaces during pen presentations from previous experimental runs. The signal at *m/z* 57 from *n*-butanol during pen presentation was subsequently corrected for this noise via subtraction of the respective mean value for each measurement series. This background noise signal was additionally used to calculate the PTR-MS limit of detection (LOD) for gas-phase *n*-butanol for each measurement series based on three standard deviations of the mean noise signal. Correction factors were then applied to the data to account for the abundance of the *n*-butanol fragment at *m/z* 57 and the *m/z*-dependent transmission efficiency of the mass spectrometer. A gas-phase VMR, or concentration^1^ of *n*-butanol was subsequently calculated from a theoretical approach using the measured and isotope-corrected abundance of hydronium (at *m/z* 21) and a rate constant of *k* = 2.47 × 10^−9^ cm^3^ s^−1^ of the proton transfer reaction between hydronium and *n*-butanol reported in the literature (Zhao and Zhang 2004). The calculated concentrations for each measurement series were then filtered according to individual pen presentations using the flag of the manually triggered electronic analog signal. From these data, the maximum VMR during each pen measurement was calculated, and a mean (and standard deviation) of the three maxima (of triplicate measurements) was calculated for each pen no. and test, as are presented in the ensuing results and discussion.

### Statistical Analysis

Measured gas-phase concentrations of *n*-butanol were transformed to log_2_ units. Within each measurement condition, the replicate values for each pen were averaged and the results plotted against the aqueous-phase *n*-butanol concentration of the pens (also in log_2_ units). The mean log_2_ concentration was then regressed on the log_2_ aqueous-phase concentration, separately for each set of pens (Weisberg 2013). To avoid concentrations at or below the PTR-MS LOD, these analyses were conducted using the data for pen nos. 0–12 only. A quadratic term was added to the model to test for non-linearity. Residual plots were used to evaluate the adequacy of the models.

To evaluate the effect of repeated application of the pens (see Application Test), a regression model was fitted to the log_2_ concentrations that included a linear term for application (1–6) and an indicator variable for each pen (0–16). In addition, the observed first differences between successive applications (e.g., second to first application, third to second application, etc.) were plotted separately by pen and tested for equality of means across the five differences using Hotelling’s *T* ^2^ test (Johnson and Wichern 2007).

To estimate the differences between testing conditions and between different pen sets, paired *t* tests were used (Rice 2007), with each pair consisting of the mean log_2_ gas-phase *n*-butanol concentrations for a given pen number in both sets or conditions being compared (pen nos. 0–12 only). The average difference, together with the endpoints of its 95 % confidence interval, was then exponentiated to obtain the percentage change in concentration from one condition or set to the other. Regression models were used to fit to the data from multiple conditions or sets, estimating a different intercept for each. In cases where the slopes differed between conditions or sets (as determined by introducing an interaction effect between aqueous-phase concentration and condition), the fitted model was used to estimate the difference between conditions/sets at different aqueous-phase concentrations (Weisberg 2013).

## Results

**Fig. 3:**
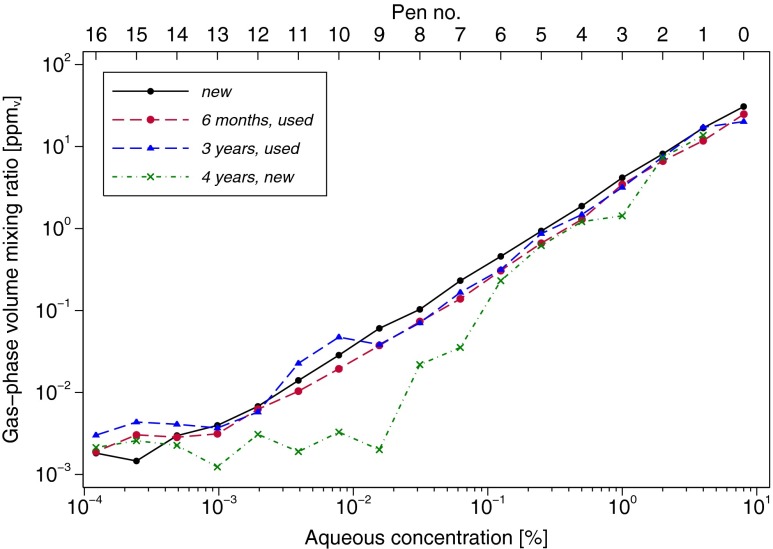
*n*-Butanol gas-phase volume mixing ratio plotted versus the aqueous-phase concentration within the pen, separately for each pen of the *new*; *6 months, used*; *3 years, used*; and *4 years, new* Sniffin’ Sticks test sets. Each point represents the average of up to three replicates, computed in log_2_ units. Due to an increase in the PTR-MS LOD from one measurement series to the next (see “Results”), some points represent only a single measurement or the average of two replicates, as follows: *new*: pen nos. 14–16 are single measurements, while pen no. 13 is the average of two replicates; *6 months, used:* pen nos. 13 and 16 are the average of two replicates; *4 years, new set*: pen nos. 9–16 are single measurements

The data from the first measurement series of the *new* set are shown in Fig. 2. The PTR-MS LOD increased slightly from one measurement series to the next (not shown; see “Validation of the Concentration Linearity” section for discussion). For example, LODs for gas-phase *n*-butanol measured during the linearity validation assessments were 0.86 ppb_v_, 4.57 ppb_v_ and 7.02 ppb_v_, respectively, for the first, second, and third measurement series of the *new* set, and 0.64 ppb_v_, 1.60 ppb_v_, and 2.93 ppb_v_, respectively, for the first, second, and third measurement series under the *6 months, used* condition. As a result, in some cases, it was not possible to obtain a valid second or third replicate for the lowest concentration pens (nos. 13–16), since the PTR-MS LOD was still elevated following analysis of the highest concentration pens from the preceding measurement series. The effect of the LOD is visible as a leveling off in the relationship between measured (gas-phase) and given aqueous-phase concentrations for pens at the lowest concentrations (Figs. 3 and 5).

### Concentration Linearity

The measured gas-phase *n*-butanol concentrations of pen nos. 0–12 in the *new* set were linearly associated with the aqueous-phase concentrations in the pens (*p* = 0.705 for a quadratic term), with an estimated slope of 1.02 (95 % CI, 1.01–1.03), nearly identical to the theoretical value of 1 (Fig. 3). Quadratic terms were also not statistically significant for the other two pen sets (*p* = 0.339 for *3 years, used* and *p* = 0.410 (pen nos. 1–8) for *4 years, new*). In contrast, there was evidence of non-linearity among the pens subjected to stress testing (*p* < 0.001 for *recapped*, *p* = 0.070 for *regeneration*, and *p* = 0.022 for *6 months, used*) except under the *uncapped* condition (*p* = 0.599, Fig. 5). However, in each case, the amount of curvature was very slight compared with the linear effect and was mainly located toward the weaker concentrations, possibly due to approaching the PTR-MS LOD. Finally, the estimated slopes for the experimental conditions, the *6 months, used* and the *3 years, used* pens were all similarly close to 1. Only the *4 years, new* pen nos. 1–8 exhibited a slope that was substantially different from 1 (1.34, 95 % CI, 1.11–1.57), including additional pens (of lower concentration), resulting in an even larger estimated slope, but these were excluded here because they are not adequately represented by the linear model (Fig. 3).

### Application Test

**Fig. 4:**
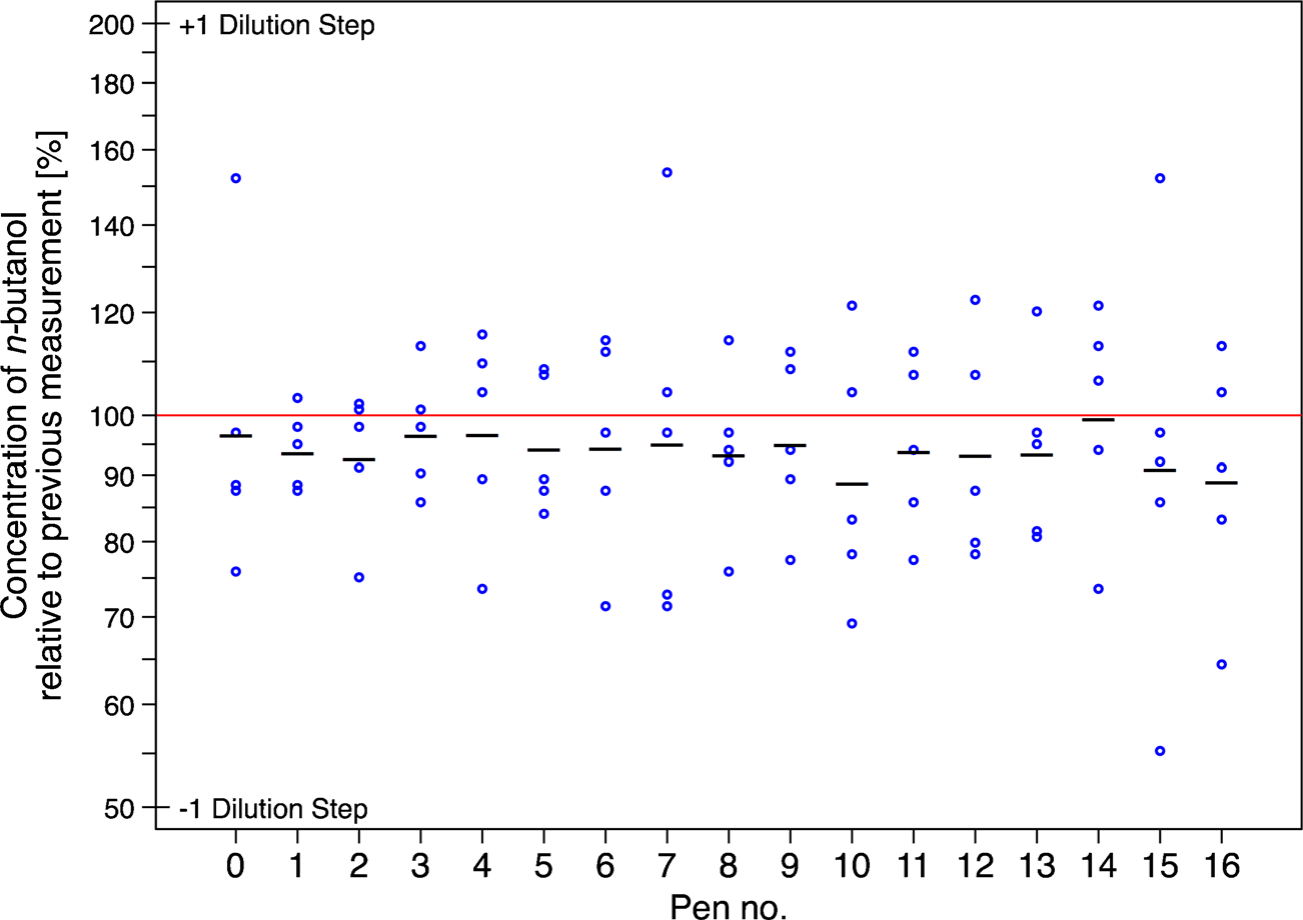
Observed changes from each of six successive measurements to the next for the application test, together with the average change for each pen. Successive measurements were obtained 20 s apart. *Plotted points* represent the first differences between each adjacent pair of measurements (e.g., 2–1, 3–2, etc.), while the *black lines* indicate the mean difference for each pen. A decrease of 50 % or an increase of 100 % correspond to a decrease or increase of a full dilution step (i.e., a whole pen step), respectively

After presenting each *new* pen six times in succession, the average slope across all 17 pens (nos. 0–16) was −0.07 log_2_ units, corresponding to a decrease in the measured concentration of 5 % (95 % CI, 4–6 %) for each successive presentation, or a total of 23 % (95 % CI, 17–28 %) over all six presentations. Figure 4 shows the difference in concentration at each presentation compared with the previous one, separately by pen. Both the means and the variability around them are considerably smaller than ±1 log_2_ unit, which would correspond to an increase or decrease of a single dilution step. Hotelling’s *T* ^2^ test applied to the five mean first differences (i.e., 2–1, 3–2, etc.) yields a *p* value of 0.436, providing no evidence that the average change differed across the six presentations.

### Effects of Pen Age

Table 2 compares the concentrations among the three pen sets, including the first set measured both as *new* and after 6 months (*6 months, used*) after the stress tests (*recapped*, *uncapped*, and *regeneration*) had been performed. The average change among the *6 months, used* pens compared with the pens when new was −0.46 log_2_ units, corresponding to a decrease of 27 % (95 % CI, 22–33 %). In contrast, the average difference between the *3 years, used* and the *new* pens was only −0.20 log_2_ units, a difference that was not statistically significant. Finally, the *4 years, new* pens were not statistically different from the *new* pens at an aqueous-phase concentration of 4 % (pen no. 1) but, at a dilution of 0.031 % (i.e. pen no. 8), were on average 2.17 log_2_ units lower than *new*, corresponding to a reduction of 78 % (95 % CI, 60–88 %).

**Table 2:** Mean difference in measured gas-phase *n*-butanol concentration between the *new* pen set and each additional set and condition evaluated

Condition*	Mean difference from *new* (log_2_)	Standard error	% Change	95 % Confidence interval
6 months, used	−0.46	0.05	−27 %	(−33 %, −22 %)
3 years, used	−0.20	0.13	−13 %	(−28 %, +6 %)
4 years, new				
Pen no. 1 (4 % *v/v*)	−0.11	0.40	−8 %	(−49 %, +68 %)
Pen no. 8 (0.031 % v*/v*)	−2.17	0.40	−78 %	(−88 %, −60 %)
Recapped	−1.35	0.06	−61 %	(−64 %, −57 %)
Uncapped	−1.34	0.10	−60 %	(−66 %, −54 %)
Regeneration	−0.55	0.04	−32 %	(−36 %, −28 %)

A change of −1 log_2_ units (i.e., −50 %) is equivalent to a decrease of a full dilution step (i.e., a whole pen). In the *4 years, new* set, single measurements only were obtained for pen nos. 9–16, all of which were at or near the PTR-MS LOD (see text for discussion). Since a regression line fit to pen nos. 1–8 yielded a slope greater than 1, estimates of the difference between this set and the *new* set are given for aqueous-phase concentrations of both 4 % (pen no. 1) and 0.031 % (pen no. 8)

^a^Pens 0–12 or 1–12, where applicable

### Stress Tests

Gas-phase *n*-butanol concentrations measured following the conditions *recapped* and *uncapped* were on average 1.35 and 1.34 log_2_ units lower than those measured when the pens were new, corresponding to decreases of 61 % (95 % CI, 57–64 %) and 60 % (95 % CI, 54–66 %), respectively (see Table 2). This corresponds to a reduction of just over one dilution step. Some recovery was evident following the *regeneration* condition in which concentrations differed on average from the *new* pens by only −0.55 log_2_ units or −32 % (95 % CI −36 to −28 %). Note that this difference is nearly identical to the difference still observed for the same pens 6 months later (*6 months, used* condition; see above), indicating that no further recovery was evident.

## Discussion

It was previously only assumed that the odor release from the tips of Sniffin’ Sticks pens behave linearly according to their aqueous-phase dilution series. Only one scientific study on the release of *n*-butanol from the Sniffin’ Sticks pens could be found in the literature. Haberland and co-workers used gas chromatographic methods to investigate the short- and long-term continuous release from the tips of individual pens but did not compare the release over the pen set (Haberland et al. 2005). Results of their study demonstrated a rapid evaporation of *n*-butanol accumulated in the tip of the pen after opening the cap. Furthermore, they verified the decreased odor intensity reported by test subjects during use of the Sniffin’ Sticks.

### Validation of the Concentration Linearity

The present measurements of the *new* set show that the gas-phase concentrations of *n*-butanol released from the tips of these pens are indeed linear over the entire range (Fig. 3). This was similarly observed for pen nos. 0–13 in the *6 months, used* condition and for the entire series of the *3 years, used* set. The slight concentration tailing observed for the weakest pens (pen nos. 14–16) was due to limitations in the analytical procedure as these concentrations approach the LOD of the PTR-MS for *n*-butanol in the present setup. As indicated in the “Results” section above, the LOD of the instrumental setup was immediately affected by measurement of the strongest pens (pen nos. 1 and 0) such that the signal response of the subsequently measured weakest pens (typically pen nos. 13–16) was below the LOD and therefore not included in the results. This can be seen clearly in Fig. 2, which displays an entire data series of one measurement run, with measurements starting at pen no. 16 and ending at pen no. 0. At the beginning of the measurements, the PTR-MS signals for *n*-butanol (odorant concentration) immediately drop after removal of the pen from the measurement vessel, with the signal rapidly returning to the pre-exposure (background) levels. Toward the end of the measurements, however, where more highly concentrated pens are measured (approximately from pen no. 4 onwards in Fig. 2), the signal drops display significant tailing and clearly do not reach the initial background (pre-exposure) levels, nor the concentration levels of the weakest pens, within the timeframe of this snapshot. Furthermore, at such low concentrations close to the LOD, the PTR-MS signal response is more prone to statistical fluctuations from noise and can thereby cause large relative changes in the low concentrations measured. Thus, the LOD prevents assessment of the lowest concentrations (pen nos. 13–16) (Table 1). Nevertheless, using this method the gas-phase concentrations of *n*-butanol from pen nos. 0–13 could be reliably determined. Furthermore, the standard *n*-butanol odor thresholds of healthy subjects aged 16–35 years is pen no. 9.39 ± 2.56 for female subjects and pen no. 9.24 ± 2.99 for male subjects, which is within the range of the present measurements (Hummel et al. 2007).

The release of *n*-butanol in the *3 years, used* set was linear over the entire concentration range, and absolute concentrations were comparable with those of the *new* set (Fig. 3). The lack of difference observed between the *new* and *3 years, used* set indicates that, with proper usage (compared to the stress testing) and proper storage, the concentrations of the pens do not significantly degrade, even over this period that goes well beyond the intended lifetime of the pens. When not in use, these pens were kept in an air-conditioned room at approximately 23 °C throughout the entire 3-year period. By comparison, the *4 years, new* set was stored in a non-air-conditioned office with estimated temperature variations of between ∼24 and 32 °C throughout the 4-year storage period. This might explain the large variability in the data between individual pens of this set, the non-linearity of the set, as well as the overall depletion in concentrations in comparison to the *new* set, despite this *4 years, new* set being unused prior to the assessments performed here.

In relation to these findings, it should also be reiterated that the quantitation of gas-phase *n*-butanol was made by conversion of the PTR-MS signal to a concentration using an experimentally determined rate constant reported in the literature for the reaction of hydronium with *n*-butanol, as described in “PTR-MS Data Evaluation” section. This theoretical approach allows a quantitation to be made in the absence of gas standards of target compounds and obviates the need for calibration under certain circumstances (Beauchamp et al. 2013). One drawback of this theoretical approach, however, is that it does not take into account instrument-specific nuances in the sensitivity of the detection system, specifically the secondary electron multiplier (SEM), which exist between detectors of different manufacturers or batches, or during aging of the detector. Such effects cannot be accounted for without accurate calibration of the instrument. It should therefore be noted here that the concentrations determined in the present study for the measurements of the *new* and *6 months, used* conditions of the same set of pens might thus be affected by such variations: Any potential deterioration in SEM sensitivity over this period would lead systematically to lower concentrations of the pens in the *6 months, used* condition compared with the *new* set. As such, although the lower concentrations observed for the *6 months, used* condition are likely to predominantly relate directly to actual physical losses of *n*-butanol in the pens, a small contribution of detector sensitivity deterioration to the diminished absolute values cannot be ruled out entirely. This potential influence is only relevant for these specific measurements that took place 6 months apart, i.e., the direct comparison between the *new* and *6 months, used* conditions, and not for the other assessments reported here.

### Application Test

During olfactory sensitivity testing, a single pen is routinely presented twice in succession (Hummel et al. 1997). The present data indicate that, on average, the decrease in gas-phase *n*-butanol released from the tips of the pens between the first and second presentations is only 5 %. Given that individual pens are typically only presented twice in immediate succession during odor threshold tests, the standard clinical use of these pens should not be affected.

By comparison, there are some limited instances where a single pen might be presented more than two times in succession, for example, when the subject’s odor threshold corresponds to the weakest or strongest odor pens (pen nos. 16 and 1, respectively). The resulting concentration losses could potentially make the test harder for subjects, leading to a small underestimation in individual ability to detect a given concentration. It is therefore useful to know if the concentration of *n*-butanol released from a given pen remains constant over repeated presentations. However, the 23 % decrease observed here after the unlikely occurrence of six repeated presentations was still only the equivalent of half a dilution step (i.e., half a pen interval).

A further notable observation from the repeated presentations application test is the occasional concentration outliers within measurements of single pens. In some instances, the measured concentration during repetitions of a single pen was up to 45 % higher than the initial concentration. This effect might have arisen due to the presence of liquid droplets (of the aqueous-phase *n*-butanol solution) at the tip of the pens, which were occasionally visually observed. Exposure of these concentrated droplets to the high gas flow through the measurement chamber is expected to result in evaporation of greater amounts of *n*-butanol than from the tip itself. The presence of droplets on the tips of the pens should therefore be avoided during their clinical use, although dynamic gas flows directly at the tip of a pen during sniffing are expected to be different to those experienced in the present setup, thus the evaporation of *n*-butanol might therefore be less pronounced.

### Effects of Pen Age

Overall, the stability of the pens can be considered acceptable over their recommended 6-month lifespan. There was no significant reduction in concentration when comparing the *new* pens and the *3 years, used* pens (Table 2), nor any evidence of a reduction over the months following the *regeneration* condition (estimated change from *regeneration* to *6 months, used* condition is +5 % with 95 % CI (−4 %, +15 %)). Although the *3 years, used* pens were used only intermittently during that time, their similarity to the *new* set suggests that the pens may be stored in a controlled environment beyond their 6-month advertised lifetime without compromising their accuracy. Further studies are needed to determine how an extended period of routine clinical use may affect concentration strength.

### Stress Tests

**Fig. 5:**
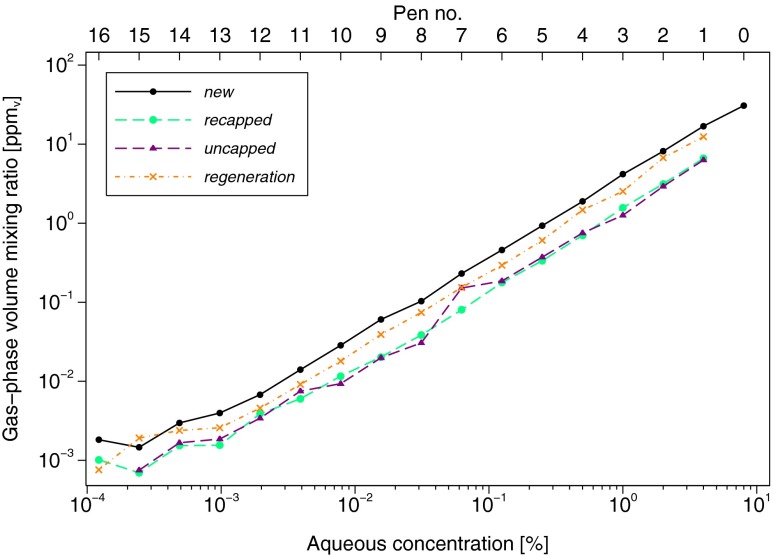
*n*-Butanol gas-phase volume mixing ratio plotted versus the aqueous-phase concentration within the pen, separately for each pen of the *new; recapped; uncapped;* and *regeneration* conditions. Each point represents the average of up to three replicates, computed in log_2_ units. Due to an increase in the PTR-MS LOD from one measurement series to the next (see “Results”), some points represent only a single measurement or the average of two replicates, as follows: *new*: pen nos. 14–16 are single measurements, while pen no. 13 is the average of two replicates; *recapped*: pen nos. 13–16 are single measurements, while pen no. 12 is the average of two replicates; *uncapped*: all points represent single measurements; *regeneration*: pen nos. 13–16 are single measurements

The results of the recapping stress test demonstrated a clear reduction in the release of gas-phase *n*-butanol over the entire dilution series in comparison to the *new* set (*recapped* vs. *new* in Fig. 5). These findings indicate that the pens are sensitive to repeated openings in quick succession. During the opening of the pen cap, *n*-butanol evaporates from the tip of the pen, and the short period of recapping was insufficient for complete regeneration of *n*-butanol at the tip of the pen.

Similarly for the second stress test, whereby the pen was left uncapped for 1 min prior to measurement (*uncapped* test), there was a clear decrease in the release of *n*-butanol over the dilution series compared with the *new* set (Fig. 5). These findings demonstrate that the pens were sensitive to longer periods of being left uncapped. The results of the test involving 4 h regeneration after uncapping (*regeneration* test) showed that the release of *n*-butanol was weaker compared with the *new* set, indicating that this regeneration period was not sufficient to replenish gas-phase concentrations to those recorded for the *new* pen set and that the pens were likely permanently damaged, given that they could not regenerate to their initial strength even after 6 months (Table 2). During normal use, the stress tests simulated here are uncommon. However, they demonstrate that inappropriate use reduced measured concentrations by a substantial amount.

### Limitations

It should be noted that the present study focused on the compound *n*-butanol and that the performance of Sniffin’ Sticks pens containing different odorants may differ depending on the physicochemical properties of the odorants, such as volatility, hydrophobicity, solubility, etc. For instance, an odorant with low volatility is expected to elicit a lower overall gas-phase concentration at the tip of the pens but consequently might offer an extended lifespan of the pens. In contrast, highly volatile compounds would be released quickly and in high amounts, which might reduce shelf-life.

## Conclusions

The present study investigated the release of *n*-butanol from the Sniffin’ Sticks threshold pens on a chemical–analytical basis via online measurement of gas-phase *n*-butanol directly at the pen tips. Measurements were made for a number of different scenarios in an attempt to simulate both routine and extreme uses of the pens. Results showed that the gas-phase concentration of *n*-butanol released from the tips of a new set of pens was linear over the entire range. This is consistent with the observations of clinical-based studies (Hummel et al. 1997; Croy et al. 2009; Haehner et al. 2009b) in which the application reliability of the test was analyzed and confirmed. The present experiments further demonstrated that the gas-phase *n*-butanol concentrations for the *6 months, used* condition and the *3 years, used* pen set were also linear. An older, unused set (*4 years, new*) that had not been appropriately stored in a climate-controlled setting showed large variability, particularly at the lower concentrations (higher pen nos.), and would not have been suitable for clinical use.

Application testing designed to mimic clinical use demonstrated that a single pen did not suffer substantial decreases in concentration between its first and second presentation. Finally, stress tests designed to mimic improper use indicated that the gas-phase *n*-butanol concentrations released from the tips of the pens cannot be completely regenerated after a period of 4 hours with the caps of the pens in place and remained similarly affected after 6 months.

Overall, a *new* set of the *n*-butanol Sniffin’ Sticks threshold evaluation test battery demonstrated excellent linearity in gas-phase odorant release over the entire range of pens and reflected the expected performance that was previously only presumed. Based on these data, it can be concluded that the pen set containing *n*-butanol is an appropriate tool for measuring olfactory sensitivity, with the caveat that the pens should be handled and stored correctly to maintain their steady performance. The results of the present investigation on *n*-butanol demonstrate the applicability of this analytical method for validating pen sets with other odorants, provided the physicochemical properties of a given test odorants are conducive to these measurement techniques.

## Abbreviations

PTR-MS: Proton-transfer-reaction mass spectrometry
UPSIT: University of Pennsylvania Smell Identification Test
CCCRT: Connecticut Chemosensory Clinical Research Test
VOCs: Volatile organic compounds
GC-MS: Gas chromatography–mass spectrometry
*v/v*: Volume per volume
ppm_v_: Parts per million, by volume
ID: Inner diameter
OD: Outer diameter
*m/z*: (Ion) mass-to-charge ratio
PFA: Perfluoroalkoxy (Teflon®)
cps: Counts per second
LOD: Limit of detection
VMR: Volume mixing ratio
sccm: Standard cubic centimeters per minute
SEM: Secondary electron multiplier

## Footnotes

1 For ease of discussion, the term “concentration” is used synonymously with gas-phase VMR.
